# TaSYP137 and TaVAMP723, the SNAREs Proteins from Wheat, Reduce Resistance to *Blumeria graminis* f. sp. *tritici*

**DOI:** 10.3390/ijms24054830

**Published:** 2023-03-02

**Authors:** Guanghao Wang, Xiangyu Zhang, Huan Guo, Chenxu Zhao, Hong Zhang, Chunhuan Chen, Wanquan Ji

**Affiliations:** 1 State Key Laboratory of Crop Stress Biology for Arid Areas, College of Agronomy, Northwest A and F University, Yangling, Xianyang 712100, China; 2 Shaanxi Research Station of Crop Gene Resources and Germplasm Enhancement, Ministry of Agriculture, Yangling, Xianyang 712100, China

**Keywords:** wheat, powdery mildew resistance, *TaVAMP723*, *TaSYP137*, gene function

## Abstract

SNARE protein is an essential factor driving vesicle fusion in eukaryotes. Several SNAREs have been shown to play a crucial role in protecting against powdery mildew and other pathogens. In our previous study, we identified SNARE family members and analyzed their expression pattern in response to powdery mildew infection. Based on quantitative expression and RNA-seq results, we focused on *TaSYP137*/*TaVAMP723* and hypothesized that they play an important role in the interaction between wheat and *Blumeria* graminis f. sp. *Tritici* (*Bgt*). In this study, we measured the expression patterns of *TaSYP132*/*TaVAMP723* genes in wheat post-infection with *Bgt* and found that the expression pattern of *TaSYP137*/*TaVAMP723* was opposite in resistant and susceptible wheat samples infected by *Bgt*. The overexpression of *TaSYP137*/*TaVAMP723* disrupted wheat’s defense against *Bgt* infection, while silencing these genes enhanced its resistance to *Bgt*. Subcellular localization studies revealed that *TaSYP137/TaVAMP723* are present in both the plasma membrane and nucleus. The interaction between *TaSYP137* and *TaVAMP723* was confirmed using the yeast two-hybrid (Y2H) system. This study offers novel insights into the involvement of SNARE proteins in the resistance of wheat against *Bgt*, thereby enhancing our comprehension of the role of the SNARE family in the pathways related to plant disease resistance.

## 1. Introduction

SNARE proteins are core factors that drive vesicle fusion in eukaryotes [1,2,3]. The SNARE family of genes can be divided into target membrane Q-SNARE and vesicular R-SNARE by their core functional domains and subcellular localization differences [1,2,3,4]. Q-SNARE is subdivided into four categories by different core functional domains: Qa-SNARE, Qb-SNARE, Qc-SNARE, and Qbc SNARE [1,2,3,5]. When the SNARE protein performs its biological function, it forms different SNARE protein complexes for specific functions [1,2,3,6]. For example, the ternary complex, composed of three different SNARE proteins, plays a role in exocrine secretion [6,7,8,9]. In contrast, the quaternary complex, composed of four SNARE proteins, acts on the fusion of intracellular vesicles [9,10].

Penetration resistance is a critical component of the host’s immune response against fungal pathogens. The process mentioned above is considered an efficient and rapid signal transduction mechanism in plants, enabling them to defend against fungal pathogen invasions. The host’s immune response to fungal pathogens involves the rapid reaction and activation of papillae to prevents fungal penetration into plant cells [11,12]. A dome-shaped cell wall is deposited between the cell wall and the plasma membrane of the penetrated part of the epidermal cells [13]. In the non-host interaction of *Arabidopsis thaliana*, non-host resistance has been proven to be mediated by the protein complex composed of PEN1, SNAP33, and VAMP721 [14,15,16,17]. In addition, *SYP121* can resist powdery mildew invasion in tomato [18]. Similarly, the wheat SNARE protein NPSN11 mediates wheat resistance to stripe rust [19]. In conclusion, SNARE proteins play a critical role as host defense signals against fungal pathogens.

Previous studies have shown that SNARE family members are involved in the plant’s response to biotic and abiotic stresses [18,20,21,22,23,24,25]. *VAMP721/722* is resistant to oomycete infection in plants and plays a role in plant growth, cell division, and abiotic stress response [26,27,28,29,30]. *SYP132* can drive hormone-regulated internal digestive transport and inhibit the density and function of the plasma membrane (PM) H^+^-ATPase [31,32]. In response to bacterial pathogens, it can promote the secretion and transportation of resistance bacteria (PR)-related proteins. These processes seem to have opposing functions, but they trigger a mechanical connection between two possible independent membrane transport pathways [32]. In addition, the overexpression of *SYP132* can inhibit bacterial invasion through the stomatal pathway [32,33]. However, when bacteria successfully bypass the stomatal defense, the overexpression of *SYP132* enhances bacterial infection [32].

While a total of 64 wheat powdery mildew resistance genes have been named and published [34], only genes at 13 loci have been successfully cloned. These genes have been identified through two main approaches: forward and reverse genetics. Mutant sequencing led to the cloning of *Pm1a*, *Pm2*, and *Pm4* [35,36,37], while map cloning was used to find *Pm3b*, *Pm5e*, *Pm24*, *Pm38*, *Pm41*, *Pm46*, and *Pm60* [35,37,38,39]. Homologous cloning was employed to identify *Pm8*, *Pm17*, and *Pm21* [40,41].

In terms of defense response to *Bgt* infection, H_2_O_2_ accumulates at the *Bgt* penetration site in *RLK* overexpressing wheat, with *TaRLK1*/*TaRLK2* potentially involved through SA and ROS [42]. Reducing the expression of *TaBON1* or *TaBON3* through virus-induced gene silencing (VIGS) can enhance wheat resistance to *Bgt* [43]. Similarly, *MLA* genes have been found to positively enhance disease resistance in both barley and wheat [44], while *TaJAZ1* [45], *TaNAC6*s [46], *TaRLK-V* [47], *TaEDS1* [48], and *TaRPP13L1-3D* [49] have also been shown to positively regulate disease resistance. On the other hand, *TaMED25* [50], *TaATG6* [51], and *TuMYB46L* [52] are among the negatively regulated disease resistance genes.

In this paper, the expression patterns of *TaSYP132/TaVAMP723* genes in wheat were measured post-infection with *Blumeria graminis* f. sp.* Tritici*, known as powdery mildew, to elucidate wheat–*Bgt* interactions. Concurrent overexpression and virus-mediated gene silencing were used to provide evidence that the genes could inhibit wheat resistance to powdery mildew invasion while a yeast two-hybrid model provided evidence of their interaction.

## 2. Results

### 2.1. Isolation of Wheat TaSYP137 and TaVAMP723 and Characterization of the Encoding Proteins

**Figure 1:**
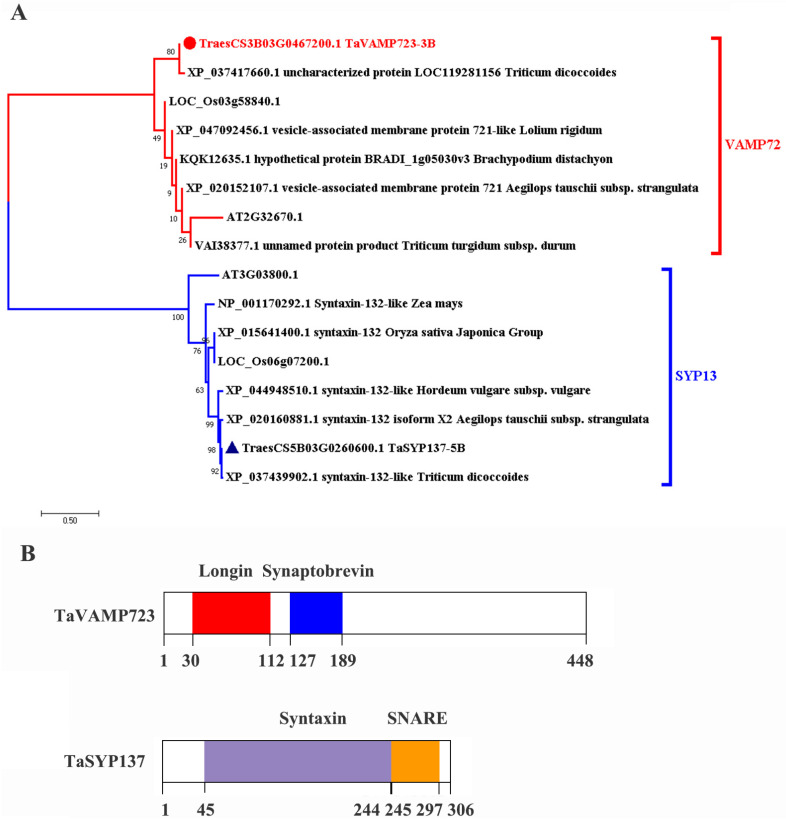
Analysis of *TaVAMP723-3B* and *TaSYP137-5B*. (**A**): Phylogenetic analysis of TaVAMP723 and TaSYP137 was conducted by constructing a phylogenetic tree using the maximum likelihood method with MEGA X software. Different SNARE proteins from *Triticum aestivum* (TaVAMP723-3B, TaSYP137-5B), *Arabidopsis thaliana* (AT2G32670.1, AT3G03800.1), *Oryza sativa* (LOC_Os03g58840.1, LOC_Os06g07200.1, XP_015641400.1), *Triticum turgidum* (VAI38377.1), *Aegilops tauschii* (XP_020152107.1, XP_020160881.1), *Brachypodium distachyon* (KQK12635.1), *Lolium rigidum* (XP_047092456.1), *Triticum dicoccoides* (XP_037417660.1, XP_037439902.1), *Hordeum vulgare* (XP_044948510.1), and *Zea mays* (NP_001170292.1). Red branches represent VAMP72-like proteins; Blue branches represent SYP13-like proteinsThe two wheat proteins TaSYP137 and TaVAMP723 are marked with blue triangle and red circles, respectively. The number on the evolutionary branches indicates the BOOTSTRAP value, and a value of 100 indicates that the probability of this branch is 100%. (**B**): The functional domains predicted by Pfam. TaVAMP723-3B has the longin domains (30 aa-112 aa) and synaptobrevin domains (127 aa-189 aa), while TaSYP137-5B contains the syntaxin domains (45 aa-244 aa) and SNARE domains (245 aa-297 aa).

*TraesCS5B03G0260600* (*XP_044387391.1*) and *TraesCS3B03G0467200* (*XP_044348247.1*) were cloned from the near-isogenic Line N9134R. The TraesCS5B03G0260600 protein contains both syntaxin domains (45 aa-244 aa) and SNARE domains (245 aa-297 aa), while the TraesCS3B03G0467200 protein consists of longin domains (30 aa-112 aa) and synaptobrevin domains (127 aa-189 aa), as shown in Figure 1B. According to the domain information, they can be identified as members of the SNARE protein family SYP13 and VAMP72, named *TaSYP137-5B* and *TaVAMP723-3B*, respectively. The full-length coding sequences (CDSs) of *TaSYP137-5B* and *TaVAMP723-3B* contain a complete open reading frame (ORF) region consisting of 307 and 449 amino acid residues (Figure 1B), respectively, with molecular weights of 35 kDa and 49.8 kDa and isoelectric points of 5.9 and 6.8, respectively (Table S1). We then extracted similar protein sequences from NCBI and other species. A phylogenetic analysis was conducted on 16 proteins belonging to the VAMP72 and SYP13 protein classes, with 8 proteins in each class, using the maximum likelihood method (Figure 1A). It was observed that the proteins shared similarities with *Triticum dicoccoides* in XP_037417660.1 and that the XP_037439902.1 protein had the closest genetic relationship (Figure 1A).

### 2.2. Expression of TaSYP137 and TaVAMP723 in Wheat-Bgt and Their Subcellular Localization

**Figure 2:**
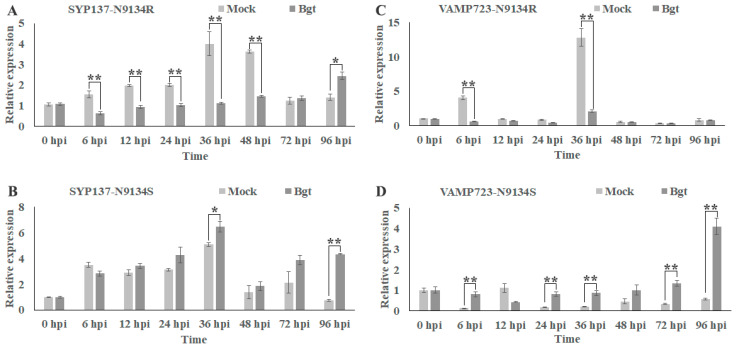
The expression patterns of *TaSYP137* and *TaVAMP723* genes were investigated in N9134R/N9134S at 0–96 h with *Bgt*/mock inoculation. *TaSYP137* and *TaVAMP723* expression levels were assessed in *Bgt*/mock inoculation-infected N9134R (resistant) and N9134S (susceptible) lines by qRT-PCR at 6, 12, 24, 36, 48, 72, and 96 hpi. Data were normalized to the β-Actin (GenBank: aK458277.1) expression level. (**A**): The expression level of *TaSYP137* gene with *Bgt*/mock inoculation in N9134R; (**B**): The expression level of *TaSYP137* gene with *Bgt*/mock inoculation in N9134S; (**C**): The expression level of *TaVAMP723* gene with *Bgt*/mock inoculation in N9134R; (**D**): The expression level of *TaVAMP723* gene with *Bgt*/mock inoculation in N9134S. Data were the mean of three biological replicates ± S.E. Asterisks denote significant differences by Student’s *t*-test analysis (*t*-tests, * *p* < 0.05, ** *p* < 0.01).

Analyzing their expression patterns in response to *Bgt* infection (Figure 2), we found that in N9134R, the expression of *TaSYP137* was significantly reduced between 6 and 48 h post-infection (hpi) with a slight recovery at 96 hpi (Figure 2A). The expression of *TaVAMP723* was also significantly reduced between 6 and 48 hpi in N9134R (Figure 2C). In N9134S, a significant increase in *TaSYP137* was seen between 24 and 96 hpi (Figure 2B), and the expression of *TaVAMP723* showed a significant reduction at 12 hpi but a significant increase at other times (Figure 2D).

**Figure 3:**
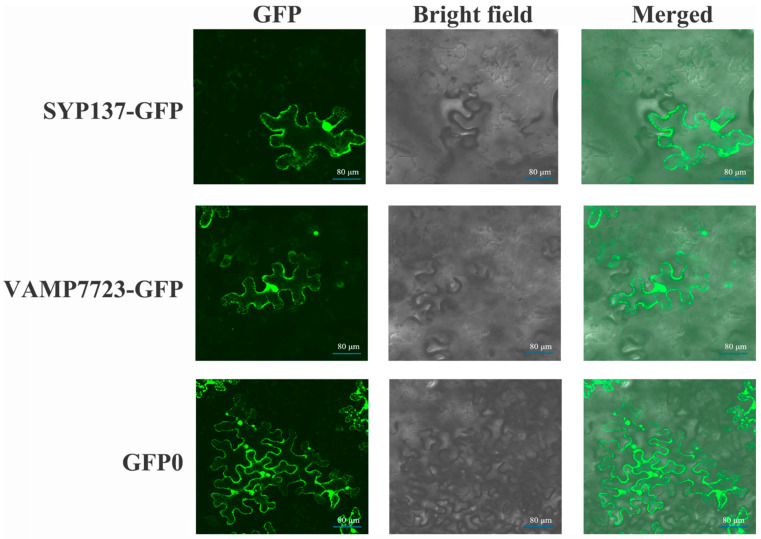
Transient expression and localization of *TaSYP137* and *TaVAMP723* fusion proteins in *Nicotiana benthamiana* non-plasmolyzed epidermal cells. The fusion vector pYJ:TaSYP137:GFP, pYJ:TaVAMP723:GFP and the pYJ::GFP control (Ck, marker with GFP0) vectors were transformed into tobacco epidermal cells by *Agrobacterium*. The subcellular distribution of GFP in the epidermal cells was revealed by fluorescence scanning microscopy.

To investigate the subcellular localization of TaVAMP723 and TaSYP137 proteins, we constructed a GFP fusion vector and transformed it into *Agrobacterium tumefaciens* (Figure 3). Then, the *Agrobacterium tumefaciens* liquid containing the recombinant vector/blank vector was injected to infest the young leaves of *Nicotiana benthamiana*. Finally, GFP fluorescence detection was performed on the epidermal cells from the abaxial side of *Nicotiana benthamiana*. The results indicated that TaVAMP723 and TaSYP137 exhibited green fluorescent signals in both the cell membrane and the nucleus. In contrast, the control GFP0 showed a wide distribution of fluorescent signals in the cytoplasm, cell membrane, and nucleus.

### 2.3. Overexpression and Silencing of TaSYP137 and TaVAMP723 Substantiated Their Negative Roles in the Response of Wheat to Bgt

**Figure 4:**
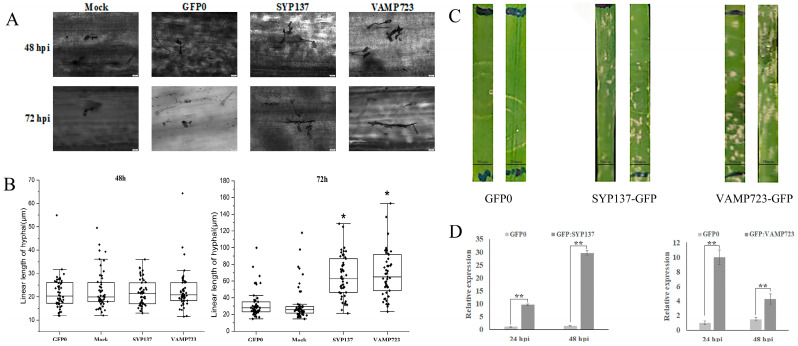
Effect of *TaSYP137* and *TaVAMP723* overexpression on the response of N9134R leaves to *Bgt* stress. (**A**): The hyphal histology pictures in the *TaSYP137* and *TaVAMP723* overexpressed N9134R leaves. (**B**): *Bgt* hyphal length after infection. (**C**): Images of infection symptoms 10 days after inoculation with *Bgt*. The reconstructed vectors, pYJ:TaSYP137:GFP and pYJ:TaVAMP723:GFP, and the pYJ::GFP control were applied to the leaves before inoculation with *Bgt* pathogen. The mock group was treated with buffer in the same way. (**D**): Relative expression levels of *TaSYP137* and *TaVAMP723*. Data were the mean of three biological replicates ± S.E. Asterisks denote significant differences by Student’s *t*-test analysis (*t*-tests, * *p* < 0.05, ** *p* < 0.01).

To explore the function of *TaSYP137* and *TaVAMP723* in the wheat–*Bgt* interaction, the genes were overexpressed in the leaves of N9134R prior to inoculation. The expression of the TaSYP137 gene was found to be upregulated by 10-fold and 30-fold at 24 hpi and 48 hpi, respectively (Figure 4D). The expression of the TaVAMP723 gene was found to be upregulated by 10-fold and 4-fold at 24 hpi and 48 hpi, respectively (Figure 4D). At 48 hpi, there was no significant difference in the mycelial length and cell size between the *TaSYP137/TaVAMP723* overexpression and control groups (Figure 4A,B). At 72 hpi, the hyphal length of *TaSYP137/TaVAMP723* overexpression was significantly longer than that of GFP0 and the control (Figure 4A,B). At 7 days post-infection (dpi), wheat leaves overexpressing *TaSYP137/TaVAMP723* showed significant spore accumulation over the control (Figure 4C). To sum up, our results indicate that *TaVAMP723* and *TaSYP137* genes were expressed at lower levels in resistant wheat and higher levels in susceptible wheat, relative to the control, when infested with the pathogen. These data provide evidence that the overexpression of *TaSYP137/TaVAMP723* inhibits wheat resistance to powdery mildew infection.

**Figure 5:**
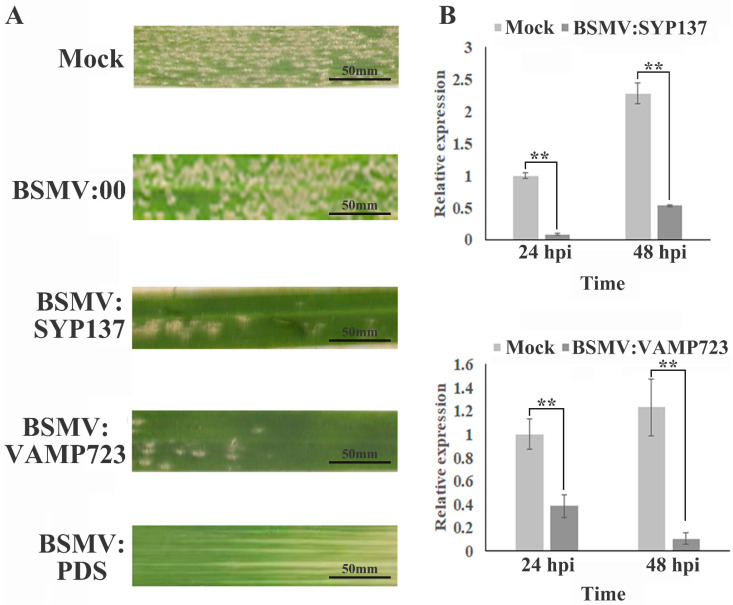
Effect of *TaSYP137* and *TaVAMP723* silencing on the response of N9134S leaves to *Bgt* stress. (**A**): Images showing the appearance of the silenced phenotype 7 days after inoculation with BSMV-PDS; the effect of the reconstructed vectors BSMV-TaSYP137 and BSMV-TaVAMP723 on inoculated leaves of N9134S leaves after *Bgt* infection; and the mock and BSMV-Blank groups treated with buffer in the same way. (**B**): Relative expression level of *TaSYP137* and *TaVAMP723*. Data were the mean of three biological replicates ± S.E. Asterisks denote significant differences by Student’s *t*-test analysis (*t*-tests, ** *p* < 0.01).

To further determine the function of *TaSYP137/TaVAMP723* in wheat’s defense against powdery mildew infection, the barley stripe mosaic virus RNA-induced gene silencing system (BSMV-VIGS) was used to silence *TaSYP137/TaVAMP723*. Phytoene desaturase (PDS) silencing was used as a proof of concept, producing albino plants (Figure 5A). Silenced *TaSYP137/TaVAMP723* plants and control plants were infected with *Bgt,* and the phenotypes were observed after 7 dpi. The results showed a significant decrease in the number and density of *Bgt* conidia in the silenced *TaSYP137/TaVAMP723* plants (Figure 5A). The qPCR assays demonstrated a silencing efficiency of 50–70% (Figure 5B). These results suggest that the suppression of *TaSYP137*/*TaVAMP723* significantly enhances wheat resistance to *Bgt.*

### 2.4. TaSYP137 Interacted with the TaVAMP723 Protein

**Figure 6:**
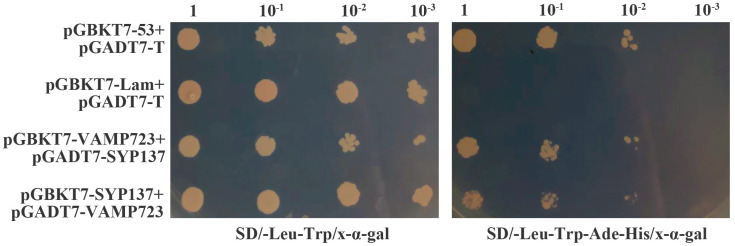
The interaction between TaSYP13 and TaVAMP723 proteins and their potential interactors in a yeast two-hybrid system. The interaction between SV40 large T-antigen (T) and murine p53 (53), T−AD + 53−BD, was used as the positive control, while the interaction between T-antigen and human lamin C (Lam), T−AD + Lam−BD, was used as the negative control.

According to previous reports, SNARE proteins form complexes to perform specific functions. In addition, SYP137 and VAMP723 can interact with each other in response to pathogenic bacteria in many other species. In this study, evidence of the interaction between TaSYP137 and TaVAMP723 was provided using the yeast two-hybrid system. We cloned *TaVAMP723* and *TaSYP137* genes into pGBKT7 and pGADT7 yeast vectors, respectively, with pGBKT7 containing the DNA-binding domain (BD) and pGADT7 containing the activation domain (AD). Positive control plasmids, pGBKT7-53 and pGADT7-T, and negative control plasmids, pGBKT7-lam and pGADT7-T, were used. For the experimental groups, pGBKT7-VAMP723 and pGADT7-SYP137 were designated as group 1, while pGBKT7-SYP137 and pGADT7-VAMP723 were designated as group 2. The yeast receptor state was transformed and then spotted on SD/-Leu-Trp to screen the transformants (Figure 6 left). The yeast solution containing transformants was then spotted on four-deficient medium SD/-Leu-Trp-Ade-His for further confirmation (Figure 6 right). The results of the study indicated that the yeast strains carrying AD-SYP137/BD-VAMP723 and BD-SYP137/AD-VAMP723 were able to grow normally on the four deficient media, indicating that the two proteins were capable of interacting in vitro (Figure 6).

## 3. Discussion

In this study, we isolated and characterized *TaSYP137*, a Q-SNARE subfamily gene, and *TaVAMP723*, an R-SNARE subfamily gene, from wheat. These genes possess distinct functional domains characteristic of their respective SNARE subfamilies. *TaSYP137* has syntaxin and SNARE domains on the C-terminal and N-terminal, respectively. *TaVAMP723* has longin and synaptobrevin domains. An evolutionary analysis of the genes TaSYP137 and TaVAMP723 shows that they are closely genetically related to XP_037417660.1 and XP_037439902.1, with high degrees of similarity to homologous proteins in other species. This suggests that TaSYP137/TaVAMP723 protein may perform similar biological functions in plant development and response to biotic and abiotic stresses, similar to the corresponding proteins in other species. It is possible to speculate on the functions of these two genes based on previous studies.

SNARE proteins play an essential role in the growth and development of all organisms [53]. The SNARE-mediated secretory pathway transfers antibacterial factors related to cell defense to the infection site during the plant’s exocytosis-related immune response [15]. *VAMP721/722* is the main exocytosis-related R-SNARE of *Arabidopsis thaliana* [54]. It is involved in many physiological processes, such as cell division, growth, biotic and abiotic stress responses, and symbiosis between plants and bacteria [15,28,29,30,55,56]. Compared with *VAMP721/722*, plant PM synthesis proteins participate in specific biological processes. Although it is unknown how VAMP721/722 participates in these biological processes, they can interact with different PM synthetic proteins, such as SYP111, SYP121, SYP122, SYP123, and SYP132, which shows that VAMP721/722 can play corresponding biological functions by interacting with the corresponding PM synthetic elements [15,28,55,56]. In previous studies, it was found that *VAMP721*/*722* played an essential role in the immune response of plants to the *Pseudomonas syringae* DC3000 bacteria [54]. The difference in the location of DC3000 bacteria, whether they are epiphytic or extracellular, could influence *VAMP721/722′*s role in bacterial immune response [54]. *VAMP721/722* is important for plant response to epiphytic bacteria, but ineffective for the immune response of bacteria that proliferate outside the cells [27,54]. Although *VAMP721* is very important for the plant immune response, there are just two associated immune factors: *RPW8.2* and phospholipid alcohol D (PLD); other directly responding immune factors are not known to be transported by vesicles of *VAMP721/722* [57,58].

Arabidopsis is a plant species that is resistant to *Blumeria graminis* f. sp. *hordei* (*Bgh*), a powdery mildew fungus, and it can resist the invasion of *Bgh* through two mechanisms: intracellular and extracellular immunity. PEN1-SNAP33-VAMP721/722 can form protein complexes to induce extracellular immunity [15]. However, *TaSYP137* and *TaVAMP723* are paralogous to *AtPEN1* and *AtVAMP721/722*, respectively. They are divided into large groups in the evolutionary tree, so *TaSYP137* and *TaVAMP723* may also form complexes to jointly perform functions in wheat. As shown in previous reports, *AtPEN1* [15], *OsPEN1* [59], *VvPEN1* [60], and *SlPEN1* [61], which belong to *SYP1*, all play a positive role in the resistance to powdery mildew, while in this study, *TaSYP137* plays a negative role, indicating that the function of genes in different species changes during differentiation.

In our earlier research, we focused on identifying the members of the SNARE family and examining their expression levels in response to powdery mildew infection. According to the quantitative expression and RNA-seq results, we focused on *SYP137*/*VAMP723* and speculated that they might play an essential role in the interaction between wheat and *Bgt* [24]. In this study, we conducted a more detailed analysis of the functions of these two genes. First, the near-isogenic Line N9134R/N9134S was used as a template for quantitative expression analysis. The expression trend of *TaSYP137/TaVAMP723* was similar. That is, the expression was downregulated in resistant and upregulated in susceptible plants at the early stage of *Bgt* induction. Further transient overexpression and silencing experiments confirmed that these genes negatively regulated wheat resistance to *Bgt* infection. Previous studies have shown that SNARE families often form complexes synergistically and that *SYP13* and *VAMP72* can interact in many other species. Therefore, yeast two-hybrid experiments were conducted in this paper to confirm that this interaction in vitro, providing evidence that *TaSYP137*/*TaVAMP723* may form a complex to negatively regulate wheat’s response to *Bgt* infection.

In summary, this research highlights that *TaSYP137*/*TaVAMP723* is present in both the nucleus and cell membrane and its expression pattern varies between resistant and susceptible wheat when infected by *Bgt*. Additionally, it was found that these two proteins can interact, which negatively regulates wheat’s resistance to *Bgt* invasion. The study of the role of SNARE in wheat resistance to *Bgt* offers new insights and expands our understanding of the role of the SNARE family in plant defense mechanisms against disease. The findings of this research demonstrate the importance of SNARE proteins in regulating the resistance of plants to fungal pathogens and contributes to a deeper understanding of the complex molecular pathways involved in plant–pathogen interactions. However, to verify the protein interactions between TaSYP137/TaVAMP723 and their function in response to powdery mildew invasion in wheat, further luciferase and wheat transgenic experiments are required.

## 4. Materials and Methods

### 4.1. Plant Materials and Pathogen Stress Treatment

The progeny of a pair of near isogenic lines, whose parents are Shaanyou225 and N9134 (containing the disease resistance gene *PmAS846*) [62], are named N9134R (resistance)/N9134S (susceptible), respectively. The near isogenic lines and their parents used in this paper are from our laboratory. The powdery mildew used was E09 (from our lab), which was stored and propagated in susceptible wheat Shaanyou225 [24]. N9134R/N9134S was incubated in a light incubator in an 16 h light/8 h dark cycle at 18 °C. Wheat plants were inoculated with powdery mildew conidia at the trilobal stage. Wheat leaves were collected at 0, 6, 12, 24, 48, 72, and 96 h after inoculation/simulated inoculation, quickly frozen in liquid nitrogen, and, finally, stored in an ultralow-temperature refrigerator at −80 °C.

### 4.2. TaSYP137 and TaVAMP723 Cloning and Sequence Analysis

PCR amplified *TaSYP137/TaVAMP723* with specific primers (Table S2) covering the whole open reading frame and using cDNA from leaves of N9134R at two days post-inoculation with *Bgt* as the template. The PCR products were purified from agarose gel and cloned into the pGEM-T Easy Vector (Promega, Madison, WI, USA) according to the manufacturer’s protocol. The nucleotide sequences of the positive clones were determined by AuGCT DNA-SYN Biotechnology Co., Ltd. (http://www.augct.com/ 28 September 2022) (Xianyang, China). The protein sequence encoded by *TaSYP137* or *TaVAMP723* was predicted using Pfam (http://pfam.xfam.org/ 28 September 2022). Sequence alignment was performed using the MEGA X software MUSCLE program, and the phylogenetic tree was constructed using the maximum likelihood method with MEGA X software (bootstrap test 1000 replicates, JTT matrix-based method).

### 4.3. Real-Time Quantitative PCR Analysis

*TaSYP137/TaVAMP723* expression profiles in infected wheat leaves of NILs were determined by real-time quantitative PCR (qPCR) analysis of cDNA samples using SYBR Green. qPCR was performed on the QuantStudio 7 Flex Real-Time PCR System (Life Technologies Corporation, Carlsbad, USA). Sequence-specific primers were designed by NCBI primer blast (https://www.ncbi.nlm.nih.gov/tools/primer-blast/ 28 September 2022) (Table S2). The amplification was conducted in a 20 μL volume according to the SYBR Premix Ex Taq manual (Takara, Dalian, China) with the following conditions: 95 °C for 30 s followed by 40 cycles of 95 °C for 5 s and 63 °C for 34 s. For each sample, reactions were carried out in triplicate, and three non-template negative controls were included. Products were analyzed by melting curves obtained at the end of the process to confirm the amplification of a single product. The standard 2^−ΔΔCT^ method was employed to quantify the relative gene expression. Mean values and standard errors were calculated with Microsoft Excel software. Student’s *t*-tests were used to analyze data with the Origin Pro program (Origin 2021b) to assess the significance of any differences between the control and treated samples or between time points, and the threshold for statistical significance was set at *p* < 0.05.

### 4.4. Vector Construction, Subcellular Localization, and Overexpression Assay

The overexpression plasmid was based on the pYJ::GFP vector driven by the Cauliflower mosaic virus (CaMV) 35S promoter (35Spro). To construct pYJ:GENE:GFP, the open reading frame sequences were ligated into the SpeI-cut plasmid using the ClonExpressII One Step Cloning Kit (Vazyme Biotech, Nanjing, China). The recombinant plasmid was transformed into *A. tumefaciens* using the freeze–thaw method. A single colony was inoculated into 50 mL liquid LB medium (50 mg/L rifampicin and 50 mg/L kanamycin) and cultured at 28 °C. The bacterial culture was then centrifuged for 10 min. For overexpression, the pellet was resuspended in suspension buffer (10 mM MgCl_2_, 10 mM MES, and 150 μm acetosyringone) to obtain an OD600 value of 0.8–1.0 according to a previously described protocol [63]. Bacteria suspended in infiltration media were injected into wheat leaves at the two-leaf stage with a syringe; leaves were injected with *A. tumefaciens* carrying the pYJ::GFP empty vector as a control. After 36 h, the wheat leaves were inoculated with *Bgt*. In our experiment, we evaluated the effectiveness of overexpression by measuring transcript levels in leaves collected at 12 and 48 h post-*Bgt* infection. The leaves that were treated with the powdery mildew fungus were also collected at 24, 48, and 72 h, and the development of spores was observed under a microscope after Coomassie brilliant blue R-250 staining. The mycelium length was determined using ImageJ software (https://cnij.imjoy.io/ 28 September 2022) [64]. *A. tumefaciens* grown in infiltration media were injected into 4-week-old *Nicotiana benthamiana* leaves, which were then grown for approximately 48 h in a growth chamber under normal conditions. The subcellular localization of 4-week-old *Nicotiana benthamiana* leaves cells was examined using a fluorescence confocal microscope with a detection wavelength of 488 nm (Nikon ECLIPSE Ti2, Tokyo, Japan).

### 4.5. Gene Silencing Induced by Tobacco Transcribed BSMV RNA in Wheat

The BSMV-VIGS was utilized to silence *TaSYP137* or *TaVAMP723*. Briefly, a fragment of the *TaSYP137* or *TaVAMP723* homolog gene isolated by qPCR was inserted into SapI-digested pCB301-BSMV-γ by homologous recombination (Clontech Inc. Palo Alto, USA). The resulting plasmid, designated γSYP137/γVAMP723, was sequenced by AuGCT DNA-SYN Biotechnology Co., Ltd. After the pCB301-BSMV-α, -β, -γVAMP723, and -γSYP137 constructs were transformed into *A. tumefaciens*, the purified *Agrobacterium* cultures α, β, and γSYP137/γVAMP723 were mixed and transformed into *Nicotiana benthamiana* to transcribe RNA in vivo. The empty γ0 vector was used as a negative control, while a construct encoding a 214 bp fragment of the wheat phytoene desaturase (PDS) gene [49], designated γPDS, was also generated. Five days after being inoculated with phosphate-diluted tissue abrasive fluids such as BSMV-SYP137, BSMV-VAMP723, BSMV blank vectors, tap water (Called the mock-BSMV inoculation), and BSMV-PDS, the second leaves of N9134R/S seedlings at the 3-leaf stage were incubated for 12 h in the dark at 22 °C with 80% relative humidity, followed by 7 days at 18–22 °C in a growth cabinet (RLD-1000D-4DW, Ningbo, China). The infected seedlings and corresponding control plants (Shaanyou225) were then challenged with *Bgt* and kept at 18–22 °C until the susceptible variety Shaanyou225 showed signs of powdery mildew. The experiment was repeated three times. To assess the efficiency of TaSYP137 and TaVAMP723 silencing, the transcript levels in leaves collected at 0, 12, and 48 hpi with *Bgt* were quantified.

### 4.6. Yeast Two-Hybrid Assays

Protein interactions among TaSYP137 and TaVAMP723 were evaluated using the MatchMaker Y2H system (Clontech Inc. Palo Alto, USA), as previously described [63]. The full-length *TaSYP137* and *TaVAMP723* ORF was amplified by PCR with specific primers with a homologous arm designed using an NCBI primer blast and ligated into the PGBKT7 plasmid (Table S2). The transcriptional activity of the transformants was evaluated by preparing ten-fold serial dilutions and using 3 μL aliquots to inoculate SD/-Leu-Trp-Ade-His medium and SD/-Leu-Trp medium containing X-α-Gal (Clontech Inc. Palo Alto, USA). The inoculated media were incubated at 30 °C for 4 days. The coding sequences were then inserted into the BD and AD vectors.

### 4.7. Statistical Analysis

All experiments conducted in this study consisted of three or more biological replicates, with each biological replicate comprising at least three technical replicates. Statistical analysis, statistical comparisons, and plotting were carried out using ImageJ and Origin Pro program. Significance analysis of the data was conducted using Student’s *t*-test, and the threshold for statistical significance was set at *p* < 0.05.

## 5. Conclusions

In this paper, we identified two interacting genes, TaVAMP723 and TaSYP137, that exert negative regulation on wheat resistance to powdery mildew invasion. The subcellular localization of TaVAMP723 and TaSYP137 is in the cell membrane, and these genes exhibit similar expression patterns in near-isogenic lines of wheat with the same powdery mildew resistance, but opposite expression patterns in near-isogenic lines of wheat with different powdery mildew resistance.

## Data Availability

Not applicable.

## Supplementary Materials

The following supporting information can be downloaded at: https://www.mdpi.com/article/10.3390/ijms24054830/s1.

## Abbreviations

*Bgt*	*Blumeria graminis* f. sp. *tritici*
*Bgh*	*Blumeria graminis* f. sp. *hordei*
BSMV	Barley stripe mosaic virus
VIGS	Virus-induced gene silencing
Hpi	Hours post-inoculation
PM	Plasma membrane
Y2H	Yeast two-hybrid
dpi	Days post-infection
